# The bigger picture of shared decision making: A service design perspective using the care path of locally advanced pancreatic cancer as a case

**DOI:** 10.1002/cam4.4145

**Published:** 2021-07-30

**Authors:** Ingeborg P. M. Griffioen, Judith A. C. Rietjens, Marijke Melles, Dirk Snelders, Marjolein Y. V. Homs, Casper H. van Eijck, Anne M. Stiggelbout

**Affiliations:** ^1^ Medical Decision Making, Department of Biomedical Data Sciences Leiden University Medical Center Leiden the Netherlands; ^2^ Faculty of Industrial Design Engineering Delft University of Technology Delft the Netherlands; ^3^ Department of Public Health Erasmus Medical Center Rotterdam the Netherlands; ^4^ Department of Medical Oncology Erasmus Medical Center Rotterdam the Netherlands; ^5^ Department of Surgery Erasmus Medical Center Rotterdam the Netherlands

**Keywords:** oncology, pancreatic cancer, qualitative, service design, shared decision making

## Abstract

**Purpose:**

Solutions to improve the implementation of shared decision making (SDM) in oncology often focus on the consultation, with limited effects. In this study, we used a service design perspective on the care path of locally advanced pancreatic cancer (LAPC). We aimed to understand how experiences of patients, their significant others, and medical professionals over the entire care path accumulate to support their ability to participate in SDM.

**Participants and methods:**

We used qualitative interviews including design research techniques with 13 patients, 13 significant others, and 11 healthcare professionals, involved in the diagnosis or treatment of LAPC. The topic list was based on the literature and an auto‐ethnography of the illness trajectory by a caregiver who is also a service design researcher. We conducted a thematic content analysis to identify themes influencing the ability to participate in SDM.

**Results:**

We found four interconnected themes: (1) Decision making is an ongoing and unpredictable process with many decision moments, often unannounced. The unpredictability of the disease course, tumor response to treatment, and consequences of choices on the quality of life complicate decision making; (2) Division of roles, tasks, and collaboration among professionals and between professionals and patients and/or their significant others is often unclear to patients and their significant others; (3) It involves “work” for patients and their significant others to obtain and understand information; (4) In “their disease journey,” patients are confronted with unexpected energy drains and energy boosts, that influence their level of empowerment to participate in SDM.

**Conclusion:**

The service design perspective uncovered how the stage for SDM is often set outside the consultation, which might explain the limited effect currently seen of interventions focusing on consultation itself. Our findings serve as a starting point for (re)designing care paths to improve the implementation of SDM in oncology.

## Graphical abstract

The service design perspective uncovered four interconnected themes. Our findings serve as a starting point for (re)designing care paths to improve the implementation of SDM in oncology.

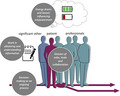

## 1 INTRODUCTION

Shared decision making (SDM) is increasingly advocated as the preferred model to engage patients in the process of deciding about diagnosis, treatment, or follow‐up when more than one medically reasonable option is available. SDM is most commonly defined as the process of integrating the best available evidence and patients’ values and preferences in the decision making process.^1^ Research has shown that SDM achieves benefits for patients and healthcare professionals.^2^, ^3^ Although SDM is acknowledged to be a process, solutions to implement SDM mainly focus on the encounter in the consultation, the training of clinicians, and the provision of decision aids to patients.^3^, ^4^, ^5^, ^6^ Despite these interventions, the effect on the implementation of SDM in oncology remains limited.^7^, ^8^, ^9^ Moreover, these solutions require extra effort from patients and oncology professionals who are already under pressure. Do we overlook the important untapped potential for improving the implementation of SDM?

We promote a new systemic perspective to expose this potential for improving SDM, that of service design. The diagnosis and treatment of patients with LAPC can be considered a service, and when co‐produced by patients and professionals (which is currently not the case) it may lead to effective SDM.^6^, ^10^ The perspective is integrative, it understands influencing factors as interconnected within the larger system of service delivery. A common approach to start a service design project is by qualitative methods to focus on the experiences with the service of those involved within a system (i.e., the patient, his or her significant other(s), and the healthcare professionals) during multiple contacts, called touch points. These touch points are interfaces between service organizations (care providers) and their clients (patients) as they occur over time, be it through material artifacts (e.g., brochures), environments, or interpersonal encounters.^11^ Understanding the service and its touch points helps to understand what people experience, what systemic influences there are on these experiences, and how these influence the ability to participate in SDM.

In this study, we used design research methods to identify the experiences of all involved in the diagnosis and treatment of locally advanced pancreatic cancer (LAPC). We used LAPC as a case, as the diagnosis and treatment of LAPC are complex and involve many decision making moments (see Box 1). Surgery is the only chance of surviving, and in case of LAPC, neo‐adjuvant chemotherapy aims to allow for surgery. Both surgery and chemotherapy carry risks of mortality and strongly impact the quality of life. Patients are made to feel that they are one of the “winners” if they qualify for surgery,^10^ hindering a weighing of the pros and cons, as is needed for true SDM.

Our aim was to understand the experiences of patients, their significant others, and their healthcare professionals during the trajectory of diagnosis and treatment that enable or hinder SDM. We use a service design perspective to understand the system causing these experiences, as the first step toward designing an improved service supporting SDM in oncology more broadly.

BOX 1 **Diagnosis and treatment of LAPC in four Dutch hospitals^12^**

The presumptive diagnosis of a LAPC is made by computed tomography (CT) scan or magnetic resonance imaging. In order to initiate treatment, the definitive diagnosis must be confirmed by a cytological or histological biopsy. In most cases, this is done with endoscopic ultrasound. To ensure that no metastasis has taken place that was not visible on the previous radiological examinations, a diagnostic laparoscopy is performed, during which a port‐a‐cath is immediately inserted for the administration of chemotherapy. Next, four cycles of FOLFIRINOX are started, followed by a CT scan for evaluation. If no progression has occurred, another four cycles of chemotherapy are administrated, and further evaluation takes place. After this, it is decided whether patients are eligible for stereotactic radiotherapy (SBRT). Before the start of SBRT, radiological fiducials will be placed endoscopically. During five consecutive days, patients will then undergo radiotherapy. Six to eight weeks after finishing this treatment protocol a new radiologic evaluation takes place to decide whether patients are suitable for an explorative laparotomy and subsequent resection of their previously inoperable tumor.

## 2 PARTICIPANTS AND METHODS

**FIGURE 1:**
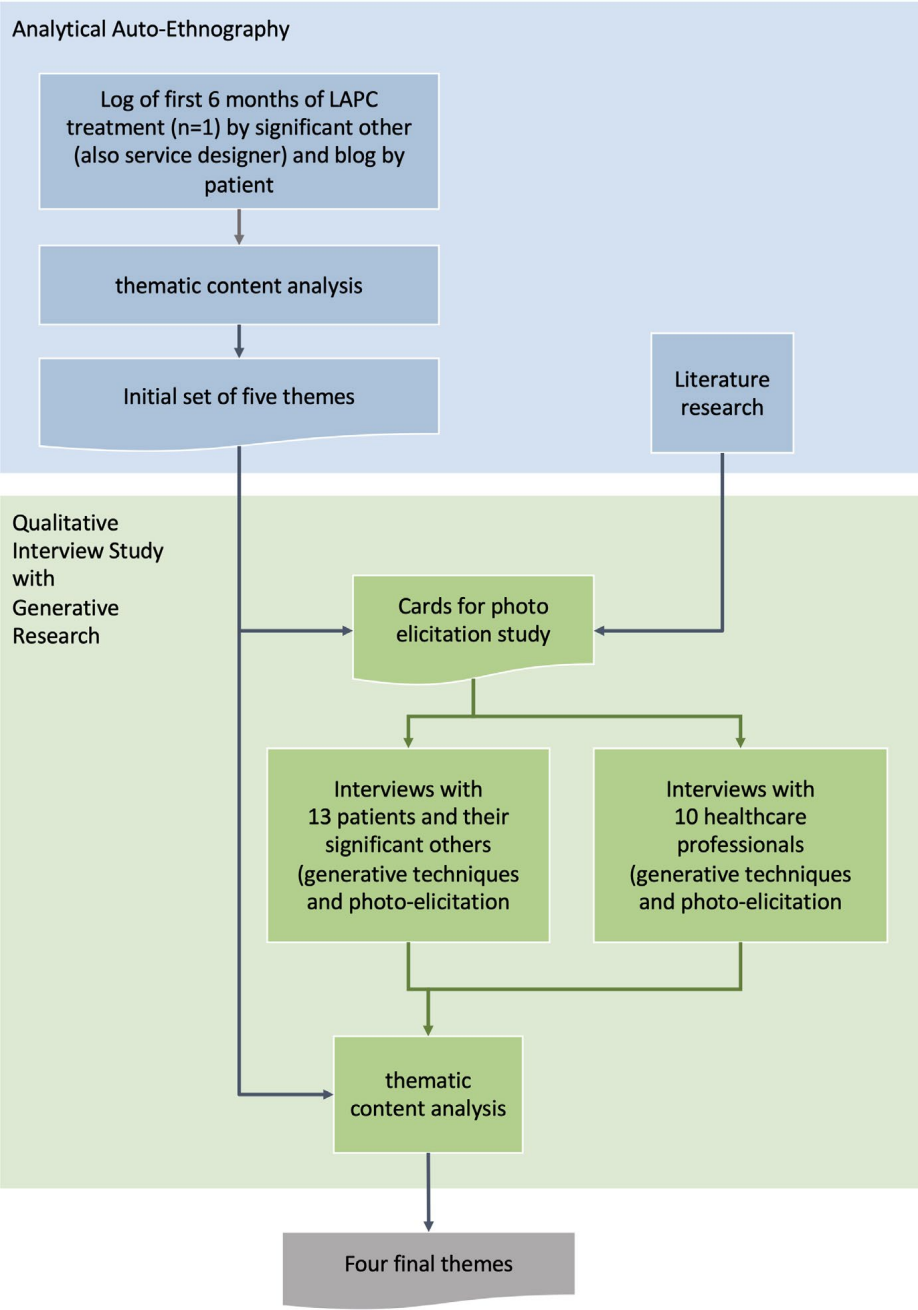
Research process

For an overview of the methods used, see Figure 1.

### 2.1 Auto‐ethnography

The first author (I.P.M.G.) is a service designer and service design researcher, who was already investigating improving the implementation of SDM when confronted with the diagnosis of LAPC of her husband. The auto‐ethnographic study^13^, ^14^ started with a log that she kept for 6 months from the start of her husband's symptoms till the end of eight courses of FOLFIRINOX and his blog. It provides both the perspective of a close significant other and that of a service designer and researcher.^15^, ^16^ The log was open‐coded by all three researchers to generate as many potential categories of codes as possible. The coding process^17^ focused on the experiences that might influence participation in SDM. The next step was axial coding to find relationships between codes. In mutual consultation, the three investigators grouped the axial codes into overarching themes (selective coding) that characterized the most important (groups of) barriers/facilitators.

### 2.2 Qualitative interview study with patients and their significant others

Subsequently, we interviewed Dutch‐ or English‐speaking patients who were treated for LAPC at Erasmus Medical Center Rotterdam, in the Netherlands, and their significant others, between February and April 2019. We continued interviewing patients until we reached the saturation of data. If possible, the patients were interviewed together with a significant other who had been present at least half of the appointments in the hospital. The majority of the interviews (11 of 13) were conducted at home, because this enabled participants to show the investigator photo's, medical devices, information, or other products that helped them to build their story.^18^ Two of the 13 patients preferred an interview in the hospital.

The content of the interviews built on the themes that resulted from the auto‐ethnography. We used design research techniques^18^: timelines^17^, ^19^ and photo‐elicitation.^20^ First, participants were asked to map their trajectory and experiences over time along a line drawn by the researcher on a roll of paper. Next, they were shown 16 cards with concepts and images and were asked whether these brought back memories of the treatment trajectory. These techniques provide instruments of thought and tools for communication to bring to the surface intuitive responses and tacit knowledge concerning the experiences with the touch points. Participants may find it difficult to express their experiences verbally for different reasons. Sometimes they are very emotional and have not deliberated the decisions, or they have rarely talked about their experiences.^10^ Noting down their experiences on a timeline and responding to images concerning touch points supported them in structuring their narrative and in highlighting their own important experiences.

The card set was based on three sources: literature,^21^ the logbook, and common service design touch points to help the participants to remember their experiences with the physical and social context (places in the hospital and at home). Participants were explicitly asked what decisions they made, what considerations played a role, and how they experienced these decision moments.

### 2.3 Qualitative interview study with professionals

**FIGURE 2:**
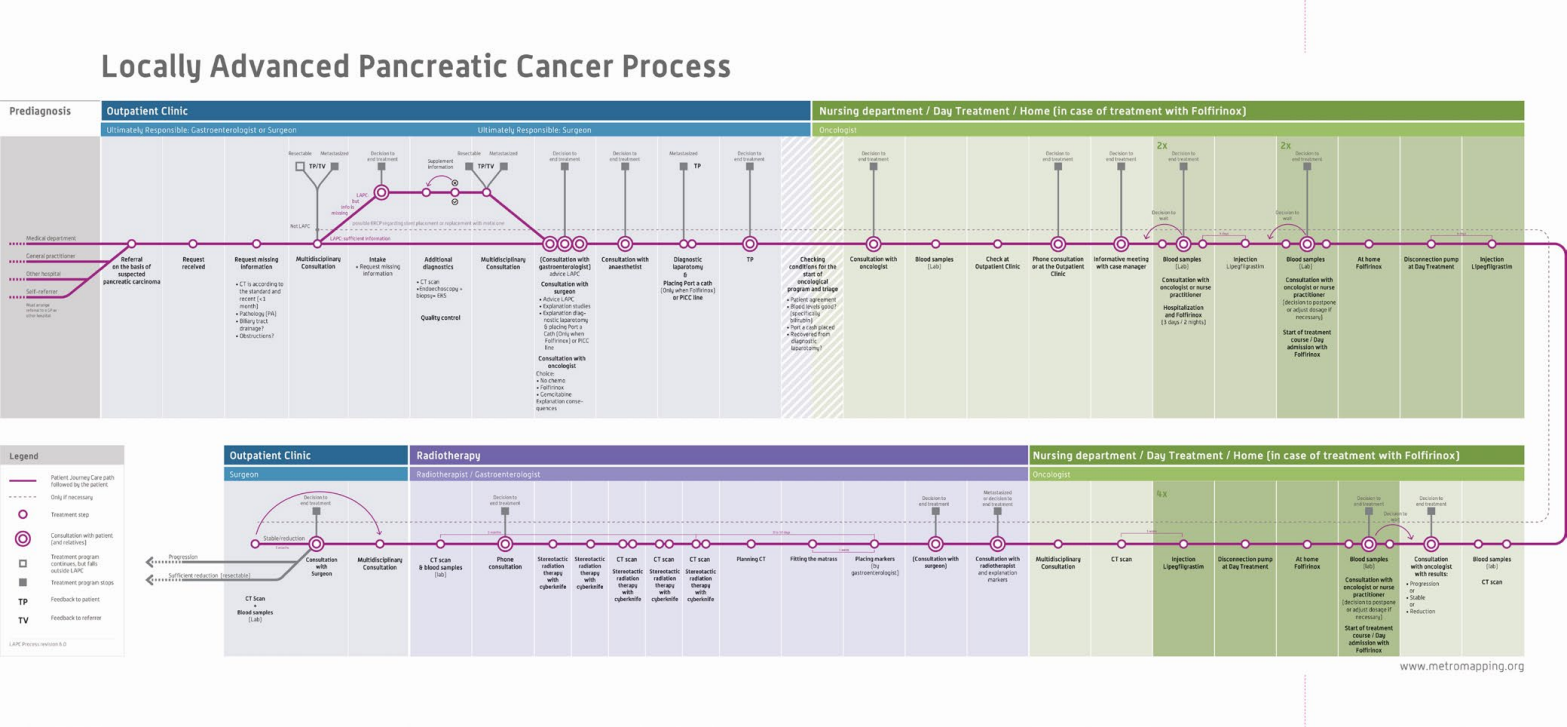
Medical metro line

We held semi‐structured interviews with healthcare professionals who have an important role in the diagnostic and treatment trajectory of patients with LAPC at the Erasmus Medical Center. The interviews also included generative techniques. We used a medical metro line (i.e., visualization of the treatment path based on the treatment protocol), as a tool for constructing the story of experiences^19^, ^22^ (Figure 2). They were also asked to respond to a set of 12 cards based on the same sources.

### 2.4 Thematic content analysis

All interviews were transcribed and anonymized. We performed a thematic content analysis.^17^ We started by open coding the first three interviews, searching for codes in line with the themes of the auto‐ethnography, framed non‐judgmentally, to avoid seeking the confirmation of the experiences described in the log. The next step was coding all interviews and axial coding to find relationships between codes. In the last step (selective coding) the three investigators defined the axial codes in mutual consideration and grouped them into overarching themes. The themes were shared for confirmation with the interviewed healthcare professionals. We did not share them with the patients and their significant others, because we tried to avoid confronting them with negative experiences of others (they were still in the treatment trajectory of LAPC).

All participants were informed about this study and asked for written consent. The Medical Ethics Committee of the Erasmus Medical Center provided a waiver of full ethical consent according to the Dutch Law on Medical Research with Humans.

## 3 RESULTS

### 3.1 Auto‐ethnography

From the log, the investigators identified four initial themes that characterized the main bottlenecks experienced and one that was supportive: decision making took place at several moments in the diagnosis and treatment process, both announced and unannounced, and with different care providers; the disease and its treatment were experienced as complex; there were many moments of confusion and the improvisational skills of patient and partner were demanded; collaboration with and among healthcare professionals was not always experienced as effective; there were times when both felt supported and empowered by the healthcare professionals.

### 3.2 Qualitative interview study

**FIGURE 3:**
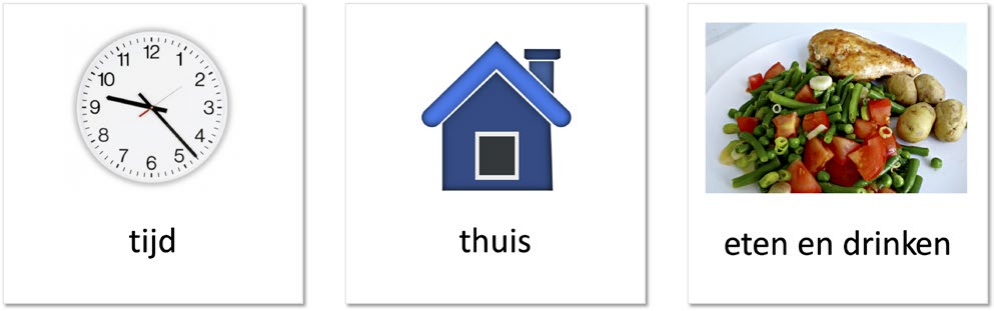
Examples of the cards (“tijd” = “time,” “thuis” = “home,” “eten en drinken” = “food and drink”)

We invited 15 patients and their significant others. Two patients refused participation, 13 were willing to participate, with their significant others. We invited 10 healthcare professionals: three surgeons, a surgical resident, a gastroenterologist, a medical oncologist, a radiation oncologist, a planning coordinator, and two specialized oncology nurses from the same academic hospital. None refused. The topics for the cards used in the interviews with patients and for the cards used in the interviews with professionals are listed in Table S1. Figure 3 shows examples of the cards.

**FIGURE 4:**
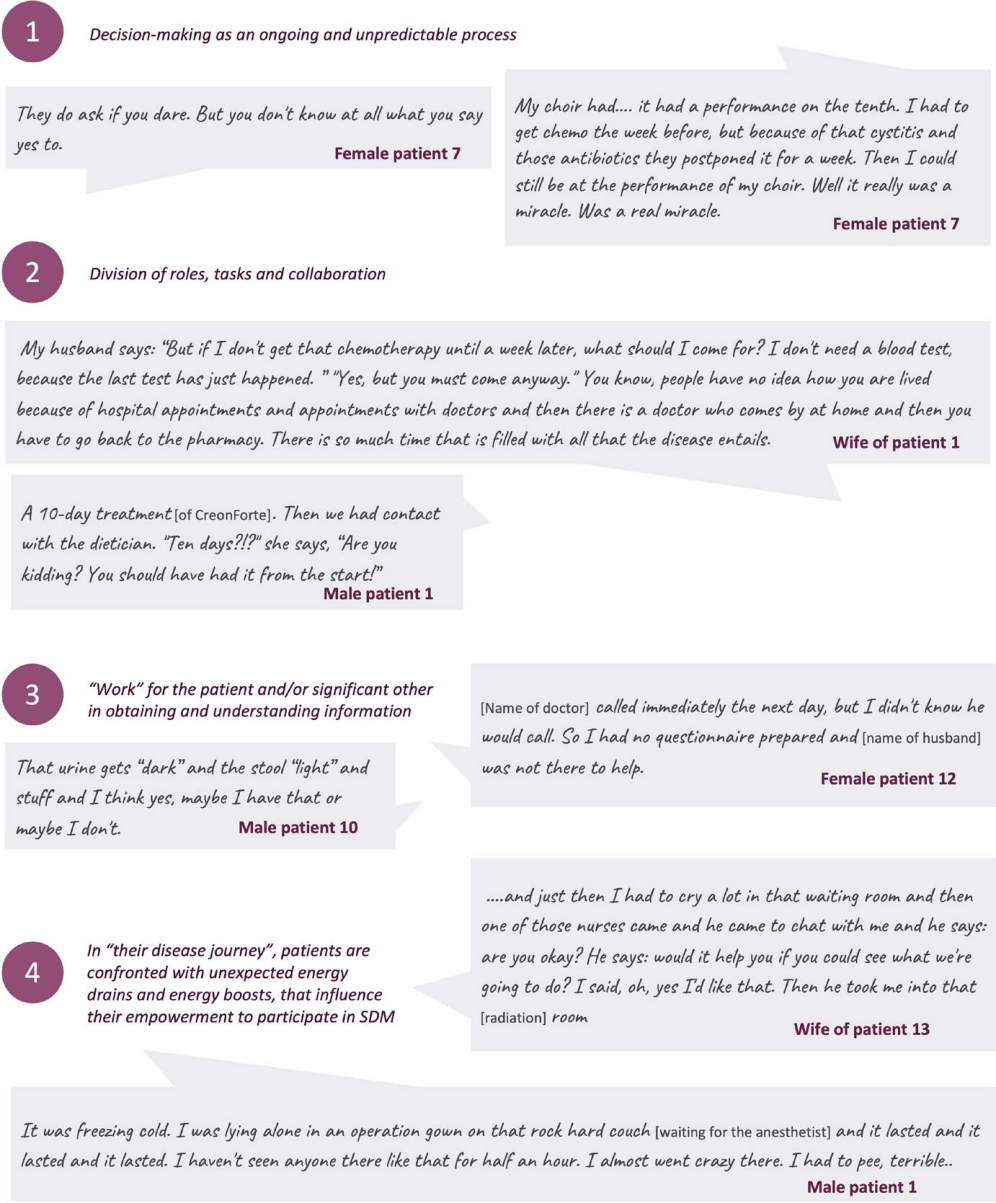
Coded quotes by patients or their significant others

Coding (see Box S1) and analysis of the interviews resulted in four final themes that describe positive and negative experiences that influence SDM. Figure 4 contains some quotes of patients or their significant others related to each theme.

1. *Decision making as an ongoing and unpredictable process*: Patients experienced that they had to make many decisions. Those are treatment decisions but also about participating in trials, practical matters (changes in nutrition, managing visitors, transportation), and existential issues (such as arrangements in case they do not survive). Decision making occurred at several moments in the trajectory, not always in formal consultations, and was often unannounced. Test results were perceived by some patients as exams to “pass” rather than as a precondition for remaining options, including “doing nothing.” Healthcare professionals mentioned that sometimes it is better to delay decision making to see how cancer progresses, despite the pressure that patients sometimes feel to move forward as fast as possible in the trajectory. Unpredictability of the (side) effects of treatment, and lack of sufficient evidence, particularly for tailoring to the individual patient, made it difficult for healthcare professionals to provide a clear recommendation. They indicated they did not always sufficiently discuss this with patients. Some mentioned that they tended to present the effect or side‐effects of therapy too optimistically because they want to give hope. Some mentioned experiencing large variation in preferences for participation in decision making among patients, and between patients and their significant others.

2. *Division of roles, tasks, and collaboration*: The treatment of LAPC and many other cancer types involve a whole team of professionals including surgeon, medical oncologist, radiotherapist, specialized nurses. It was often unclear to patients and their significant others who has which responsibility and who they should contact in emergency situations. They also experienced that GPs are not sufficiently involved or kept informed. Healthcare professionals confirmed that many different professionals are involved in the treatment, some of whom rarely see the patient. Patients often did not have a complete picture of the treatment process and its location. Patients experienced that these professionals do not always agree with each other about patient life expectancy and treatment. When other hospitals were also taking care of the patients, for example, secondary care hospitals, patients, and their significant others sometimes experienced poor coordination between hospitals. As a result, they sometimes tried to organize the coordination themselves. Furthermore, the significant others experienced a considerable burden due to extra tasks and stress, and often their social network required attention. Some patients were reluctant to express doubts about the provision of care or to give suggestions for the care path for fear of a negative influence on their relationships with professionals. Some patients felt powerless in their disease and treatment and had wanted to contribute more to the trajectory themselves. Some healthcare providers were concerned whether the patient received the required support from their significant others and whether they could handle the intense situation of the illness.

3. *“Work” for the patient and/or significant other in obtaining and understanding information*: Patients and their significant others expressed a need for more information concerning the disease, anatomy, treatment options, disease course, interpretation of results, nutrition, and self‐management. Much information was provided, but it did not always meet participants’ needs for self‐management or (participation in) decision making. The discrepancy was caused by information format, language, the amount of information, the moment it was provided (e.g., after negative results). Sometimes the information disturbed the delicate balance between patients’ hope and realism. Many patients searched for additional information. Patients and their significant others valued pictures, calm explanations, written reports, and the offer to come and take a look (e.g., at the department of radiotherapy). In particular, more adequate information was needed for medication and pain management. Jargon was sometimes difficult for patients and their significant others to understand, for example, the difference between a diagnostic laparotomy, explorative laparotomy, and pancreatoduodenectomy. Healthcare professionals encountered a range of information needs: some patients had already acquired information before the consultation, some wanted to know all details of the disease, others only wanted practical information. The majority of the information provided was oral as healthcare professionals often considered the flyers, booklets or websites unsuitable. They were unsure if their information to patients was always consistent with information from other healthcare professionals. They feared that some patients do not understand when it is important to contact them, for example, in case of fever or pain.

4. *In “their disease journey,” patients are confronted with unexpected energy drains and energy boosts, that influence their empowerment to participate in SDM*: Patients and their significant others cited various instants when reality did not match their expectations, causing tension or frustration. This included the timing of the treatment or contact moments, instructions, treatment results or side effects, disease course, and the operation of medical devices or equipment (e.g., the port‐a‐cath). They suspected that miscommunication with or between healthcare providers was usually the main cause. The environment in which the treatment takes place, or the medical devices, sometimes reinforced their feelings of vulnerability and disempowerment (e.g., full parking lots at the hospital, view on corridor while getting chemotherapy, the hospital gowns, lack of private space). Their private lives changed more than they had expected. In contrast, patients and their significant others felt empowered when turnaround times were shorter than expected. They also felt empowered when there was a calmness in the encounters with healthcare professionals, when these were friendly, or unexpectedly did something personal, for example, present a gift for their birthday. Patients and their significant others also felt empowered when they had the impression that a professional treated them as a person instead of a medical case: the professional knew their name, had read the file before the consultation, called back when promised, and took the patient's personal situation into account. Energy drains for healthcare professionals were logistic problems (such as waiting time for examinations, for information from other hospitals or healthcare providers, or for the chemotherapy), lack of overview of the logistics of the trajectory, the unpredictability of the effects and side‐effects of the treatment, and unpractical or unpleasant hospital facilities. Healthcare professionals called the nice new hospital building an energy boost. The interviews all showed great motivation of the healthcare professionals to do their best for the patients and their significant others, which was acknowledged by the latter.

## 4 DISCUSSION

In this study, we used a service design perspective to understand how a series of experiences of patients, their significant others, and professionals accumulate to the patients’ ability to participate in SDM in the diagnosis and treatment of LAPC. The design tools allowed us to elicit far more factors that (dis)empower patients and their significant others than generally observed in SDM research.

In our study, patients and their significant others provided intense descriptions of stress, fear, disempowerment, and unwanted dependence on healthcare professionals. Professionals described logistic problems, lack of overview for all involved, inconsistency in information provision to patients, and a concern about the required support in the social network of some patients. These experiences can make it hard for patients and/or significant others to participate in SDM. Some of these experiences were caused by encounters (touch points) somewhere in the service other than at a consultation. It seems that the stage for SDM is often set before the moment of consultation, which might explain the limited effect currently seen of interventions on the implementation of SDM focusing on the consultation.

Various researchers promote a wider systems approach to improve the implementation of SDM. Clayman et al.,^23^ for example, promote to include roles for and actions of family members in the decision making. They advise to focus not solely on the medical encounter but on the longitudinal nature of decisions that require ongoing adherence. Each consultation should not be considered independent but as a chapter in the entire story of a person's illness. In their systematic review of patient‐reported barriers and facilitators to SDM, Joseph‐Williams et al.^24^ conclude that patients not only lack knowledge but also the power to participate in SDM. With power they mean the perceived influence in decision making encounters, such as feeling permission to participate, confidence in own knowledge, and self‐efficacy in using SDM skills. They also indicate that some barriers to SDM are not related to what happens during the consultation, but can be traced back to how the entire healthcare system is organized. It includes time available for SDM in the consultation, continuity of care, and workflow of the healthcare setting. The larger system of influencing elements leading to either effective or ineffective SDM is left largely untouched in implementation efforts.

**FIGURE 5:**
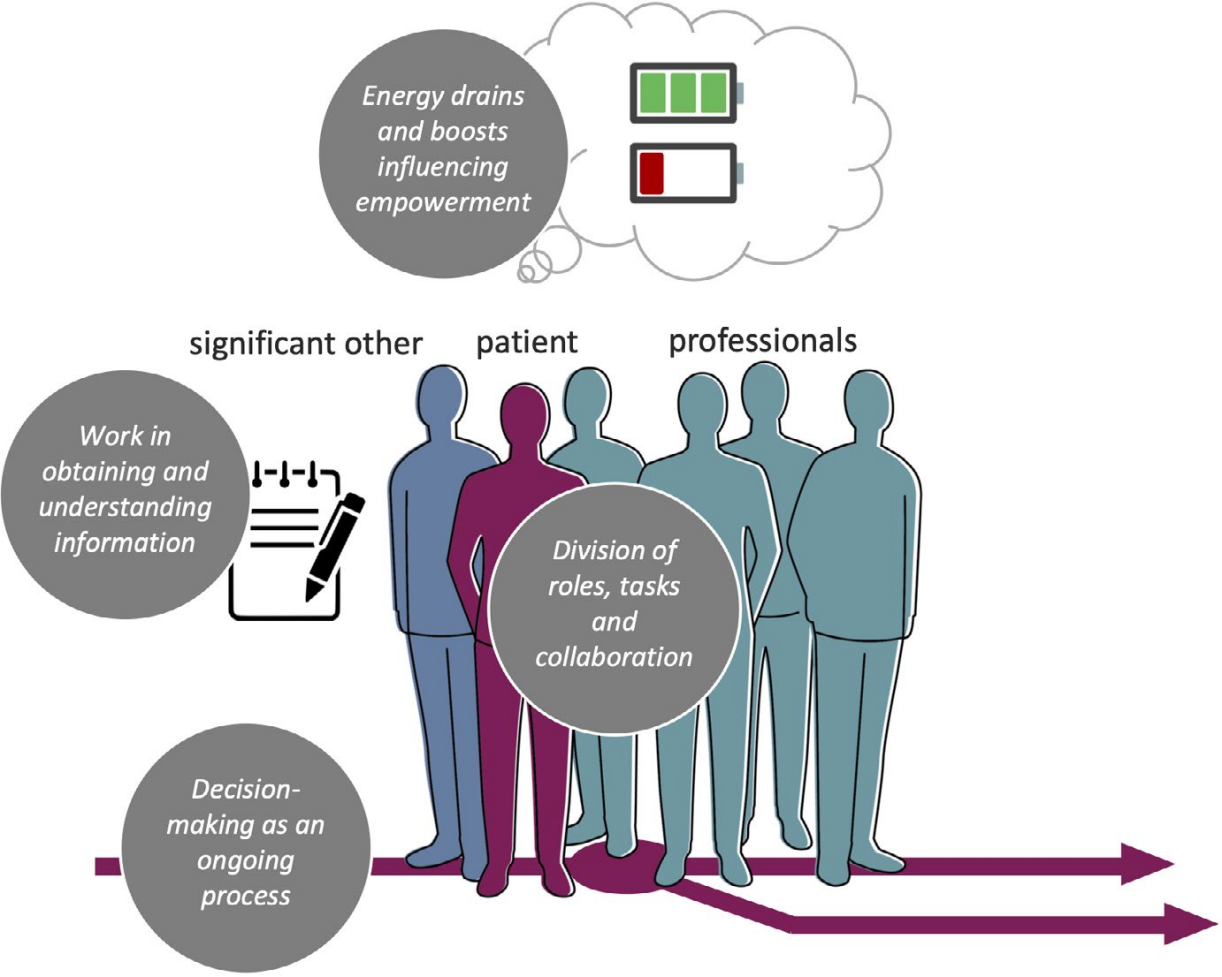
Four interconnected themes

Although the four themes we found each highlight a different aspect of the context in which SDM takes place, these aspects do not stand alone. The first three (decision making as an ongoing and unpredictable process, roles/tasks/coordination, and work for the patient) relate to organizational aspects of the care path which sometimes influenced each other: the collaboration between and with professionals sometimes led to information that was or was not shared with patients. In turn, this led to a lack of overview of the care path, including the decision moments. Various energy boost and drains mentioned within the fourth theme were often caused by the expectation of a patient, significant other, or professional regarding the first three themes that deviated from reality, in a positive or negative sense. The four themes are not separate barriers or facilitators, but care system elements that are interconnected (Figure 5).

The logfile was written by the first author, a caregiver, who is also a service designer. She also did all the interviews. This double perspective might be a limitation of the study as well as a strength. Service designers have an eye for improvement: they are trained to find potentially problematic situations as starting points for (re)design to improve a service. As a result, it could be a limitation that the first author potentially had a strong focus on the current problematic aspects of the service of LAPC which might have had an impact on both the logfile and the interviews. A potential strength is a fact that the interviewees were informed of the fact that the researcher was a fellow sufferer. It might have encouraged them to share their experiences more openly.

The service design perspective had additional benefits. First, it ensured that not only a single moment (a consultation) was considered, but it drew attention to the entire process, the service that is provided. During this service, a series of experiences is built up through touch points and this accumulated experience can lead to certain behavior. In case of limited consistency in that series of touch points, patients, their significant others, and professionals need to improvise and cope. Coping drains energy. In our research, we discovered several such experiences prior to consultations, some of which may prevent good participation in SDM.

Second, the service design perspective also sheds light on the effect of experiences with the larger context of care, such as the disempowering effect of the physical context (full parking lots at the hospital or lack of private space) and “work” in the social context (such as informing the social network of a patient and organizing visits at home).

To improve the implementation of SDM in clinical practice we recommend (re)designing the care path addressing these themes simultaneously. It would require providing a better overview of the care path and its decision making moments, a better understanding of the unpredictability of the care path, better information provision, and a clearer division of roles and tasks and improved collaboration. Stepped wedge randomized controlled trials^25^ could be the next step for researching the subsequent effects of the redesigned care paths on the implementation of SDM in clinical practice. We recommend a mixed‐methods approach, including qualitative research methods such as participatory action research^26^ to fully capture and consider the complexities of the implementation process, including dealing with differences in healthcare systems. We also recommend to further investigate, understand, and deal with the causes of the patients’ and significant others’ energy drains and energy boosts.

We believe the four final interconnected themes are not unique to LAPC or our hospital, but further research is needed to discover if these are also important in improving the implementation of SDM in the diagnosis and treatment of other cancers besides LAPC, and in other hospitals.

In conclusion, a service design perspective could be included to optimize participation in SDM for patients, their significant others, and medical professionals in the diagnosis and treatment of LAPC.

## MEDICAL ETHICS STATEMENT

All participants were informed about this study and asked for written consent. The Medical Ethics Committee of the Erasmus Medical Center provided a waiver of full ethical consent according to the Dutch Law on Medical Research with Humans.

## CONFLICT OF INTEREST

The authors indicated no potential conflicts of interest.

## Supporting information

BOX S1

TABLE S1

## Data Availability

Transcripts of the interviews can be received from the corresponding author, upon request, after our study has finished.

## References

[1] Stiggelbout AM, Pieterse AH, De Haes JCJM. Shared decision making: concepts, evidence, and practice. Patient Educ Couns. 2015;98:1172‐1179. 26215573 10.1016/j.pec.2015.06.022

[2] Kashaf MS, McGill E. Does shared decision making in cancer treatment improve quality of life? A systematic literature review. Med Decis Making. 2015;35:1037‐1048. 26246515 10.1177/0272989X15598529

[3] Shay LA, Lafata JE. Where is the evidence? A systematic review of shared decision making and patient outcomes. Med Decis Making. 2014;35:114‐131. 10.1177/0272989x14551638 25351843 PMC4270851

[4] Montori VM, Kunneman M, Brito JP. Shared decision making and improving health care: the answer is not in. JAMA. 2017;318:657‐662. 28810005 10.1001/jama.2017.10168

[5] Durand M‐A, Barr PJ, Walsh T, et al. Incentivizing shared decision making in the USA – where are we now? Healthcare. 2015;3:97‐101. 26179730 10.1016/j.hjdsi.2014.10.008

[6] Griffioen I, Melles M, Stiggelbout A, Snelders D. The potential of service design for improving the implementation of shared decision‐making. Des Health. 2017;1:194‐209. 10.1080/24735132.2017.1386944

[7] Snijders HS, Kunneman M, Bonsing BA, et al. Preoperative risk information and patient involvement in surgical treatment for rectal and sigmoid cancer. Colorectal Dis. 2014;16:O43‐O49. 24188458 10.1111/codi.12481

[8] Kunneman M, Engelhardt EG, Ten Hove FL, et al. Deciding about (neo‐)adjuvant rectal and breast cancer treatment: missed opportunities for shared decision making. Acta Oncol. 2016;55:134‐139. 26237738 10.3109/0284186X.2015.1068447

[9] Singh S, Butow P, Charles M, et al. Shared decision making in oncology: assessing oncologist behaviour in consultations in which adjuvant therapy is considered after primary surgical treatment. Health Expect. 2010;13:244‐257. 20579121 10.1111/j.1369-7625.2009.00587.x PMC5060538

[10] Ziebland S, Chapple A, Evans J. Barriers to shared decisions in the most serious of cancers: a qualitative study of patients with pancreatic cancer treated in the UK. Health Expect. 2015;18:3302‐3312. 10.1111/hex.12319 25496598 PMC5810685

[11] Secomandi F, Snelders D. The object of service design. Des Issues. 2011;27:20‐34. 10.1162/desi_a_00088

[12] Suker M, Nuyttens JJ, Eskens FALM, et al. Efficacy and feasibility of stereotactic radiotherapy after folfirinox in patients with locally advanced pancreatic cancer (LAPC‐1 trial). EClinicalMedicine. 2019;17:100200. 31891135 10.1016/j.eclinm.2019.10.013 PMC6933188

[13] Anderson L. Analytic autoethnography. J Contemp Ethnogr. 2006;35:373‐395.

[14] Ellis C, Adams TE, Bochner AP. Autoethnography: an overview. Forum Qual Soc Res 2011;12:Art. 10.

[15] Xue H, Desmet PMA. Researcher introspection for experience‐driven design research. Des Stud. 2019;63:37‐64. 10.1016/j.destud.2019.03.001

[16] Segelstrom F, Raijmakers B. Thinking and doing ethnography in service design. Paper presented at: The IASDR: Rigor and Relevance in Design; 2009; Seoul, Korea.

[17] Green J, Thorogood N. Qualitative Methods for Health Research. 4th ed. London: SAGE; 2018.

[18] Sanders EBN, Stappers PJ. Convivial Toolbox: Generative Research for the Front End of Design. Amsterdam: BIS; 2012.

[19] Mazzetti A, Blenkinsopp J. Evaluating a visual timeline methodology for appraisal and coping research. J Occup Organ Psychol. 2012;85:649‐665.

[20] Harper D. Talking about pictures: a case for photo elicitation. Vis Stud. 2002;17:13‐26.

[21] Légaré F, Ratté S, Gravel K, Graham ID. Barriers and facilitators to implementing shared decision‐making in clinical practice: update of a systematic review of health professionals’ perceptions. Patient Educ Couns. 2008;73:526‐535. 10.1016/j.pec.2008.07.018 18752915

[22] Crilly N, Blackwell AF, Clarkson PJ. Graphic elicitation: using research diagrams as interview stimuli. Qual Res. 2016;6:341‐366.

[23] Clayman ML, Gulbrandsen P, Morris MA. A patient in the clinic; a person in the world. Why shared decision making needs to center on the person rather than the medical encounter. Patient Educ Couns. 2017;100:600‐604. 27780646 10.1016/j.pec.2016.10.016

[24] Joseph‐Williams N, Elwyn G, Edwards A. Knowledge is not power for patients: a systematic review and thematic synthesis of patient‐reported barriers and facilitators to shared decision making. Patient Educ Couns. 2014;94:291‐309. 24305642 10.1016/j.pec.2013.10.031

[25] Hussey MA, Hughes JP. Design and analysis of stepped wedge cluster randomized trails. Contemp Clinical Trials. 2007;28:182‐191. 10.1016/j.cct.2006.05.007 16829207

[26] Brady GC, Goodrich J, Roe JWG. Using experience‐based co‐design to improve the pre‐treatment care pathway for people diagnosed with head and neck cancer. Support Care Cancer. 2020;28:739‐745. 31139929 10.1007/s00520-019-04877-z

