# Supplementary material for: The bigger picture of shared decision making: A service design perspective using the care path of locally advanced pancreatic cancer as a case
Source: Cancer Med. 2021 Jul 30;10(17):5907–16. doi: 10.1002/cam4.4145 (PMC8419747; doi:10.1002/cam4.4145)
Supplement: Supplementary file 1 — BOX S1 [file CAM4-10-5907-s001.docx]

Box S1 **Coding Tree**

| A Decision-making as a process  A1. Deciding on (treatment of) the patient    B Division of roles, tasks, and collaboration   1. Unclear, unspoken or unexpectedly changing division of roles in the treatment team 2. Lack of or ineffective collaboration between professionals within a healthcare institution 3. Lack of or ineffective collaboration between healthcare institutions 4. Unclear, unspoken or unexpectedly changing role distribution between patient / family and care providers (and directing role) 5. Unclear, unspoken or unexpectedly changing role distribution between patient and family member 6. Own role and responsibility unclear or different than expected or desired 7. Lack of or ineffective communication from patient / relative with healthcare provider (s) 8. Misunderstandings 9. Behavior of the care provider deviates (in a negative sense) from expectations 10. Accessibility of care providers unclear or insufficient 11. There is no permanent contact person in the hospital, unknown or insufficiently helpful according to the patient 12. Primary practitioner unclear or insufficiently effective 13. "Sick role" patient (does not dare to stand up for his / her wishes) 14. Confidence of patient / family member in healthcare professional (s) is insufficient   C Work (for patient/relative) of information acquisition, understanding and recall   1. Looking for information 2. Medication management 3. Finding your way in a physical environment 4. Understanding pancreas, pancreatic cancer and prognosis 5. Understand and monitor treatment 6. Let the patient know themselves 7. Recognizing and dealing with critical situations 8. Form and manner of information provision (can also be too much) 9. Prepare questions   D Energy drains (undermining empowerment)   1. Treatment (or communication about it) deviates from expectations 2. Information is missing, withheld, confusing or contradictory 3. Lack of customization 4. Uncertainty, confusion and tension due to the physical environment 5. Changes in the home situation are confusing or cause tension 6. Uncertainty, confusion and tension about nutrition and losing weight 7. Uncertainty, confusion and tension about the duration or planning of the treatment 8. Uncertainty, confusion and tension as a result of hope and fear, future or prognosis 9. Uncertainty, confusion and tension about pain management and side effects 10. Lack of opportunities to stay yourself and gain privacy 11. Uncertainty, confusion and tension about medical devices (equipment or disposables) 12. Coping in response to ambiguity, confusion and tension: justify or deny   E Energy boosts (promoting empowerment)   1. Windfalls and / or positive experience of treatment duration and planning 2. Windfalls and / or positive experience of the physical environment 3. Treatment by / attitude of healthcare professional (s) gives positive energy 4. Windfalls and / or positive experience of the treatment 5. Unexpected, positive and / or empowering additional services from healthcare professionals 6. The feeling of being seen by healthcare professionals as a person with their needs |
| --- |
