# Supplementary material for: The bigger picture of shared decision making: A service design perspective using the care path of locally advanced pancreatic cancer as a case
Source: Cancer Med. 2021 Jul 30;10(17):5907–16. doi: 10.1002/cam4.4145 (PMC8419747; doi:10.1002/cam4.4145)
Supplement: Supplementary file 2 — TABLE S1 [file CAM4-10-5907-s002.docx]

**Table S1 Cards used in the interviews**

| Cards for interviews with patients and relatives | Cards for interviews with professionals |
| --- | --- |
| Decisions | Decisions |
| Information | Information |
| Confusing | Confusing |
| Scary or annoying | Scary or annoying |
| Handy or pleasant |  |
| Co-operation | Co-operation |
| Places (with picture of hospital) | Places (with picture of hospital) |
| Home |  |
| Medication |  |
| Food and drink |  |
| Family |  |
| Rest |  |
| Consultation |  |
| Admission |  |
| Medical procedure |  |
| Time | Time |
|  | Patient preferences |
|  | Patient expectations |
|  | Patient self-reliance |
|  | Patient knowledge |
|  | Changes in clinical situation |
